# Profile of red blood cell morphologies and causes of anaemia among pregnant women at first clinic visit in the mount Cameroon area: a prospective cross sectional study

**DOI:** 10.1186/s13104-017-2961-6

**Published:** 2017-11-29

**Authors:** Judith K. Anchang-Kimbi, Vera Ngenwie Nkweti, Helen Ngum Ntonifor, Tobias O. Apinjoh, Hanesh Fru Chi, Rolland Bantar Tata, Eric Akum Achidi

**Affiliations:** 1 Department of Zoology and Animal Physiology, University of Buea, 63, Buea, Cameroon; 2 Department of Biological Sciences, University of Bamenda, 39, Bamenda, Cameroon; 3 Department of Biochemistry and Molecular Biology, University of Buea, 63, Buea, Cameroon; 4 Department of Molecular Parasitology, University of Buea, 63, Buea, Cameroon

**Keywords:** Red blood cell indices, Anaemia and pregnancy

## Abstract

**Objective:**

Anaemia is a serious problem in pregnancy in malaria-endemic countries. This study investigated red cell morphologies and possible causes of anaemia among pregnant women at first clinic visit. Venous blood samples from consented women were used to determine haemoglobin (Hb) levels, mean corpuscular volume (MCV) and mean corpuscular haemoglobin (MCH) using an automated haematology analyzer. Malaria parasitaemia was diagnosed by microscopy. Definitions were as follows: anaemia (Hb < 11.0 g/dl), microcytosis (MCV < 78 fl), macrocytosis (MCV > 101 fl), hypochromasia (MCH < 27 pg), microcytic hypochromia or normocytic hypochromia with anaemia [iron deficiency anaemia (IDA)], normocytic normochromia with anaemia in the absence of malaria parasitaemia (physiological anaemia of pregnancy).

**Results:**

Of the 279 pregnant women enrolled, 57% had anaemia. Malaria parasitaemia was associated with 23.3% of anaemic cases while 76.7% were non-malaria related. The distribution of red cell alterations was as follows: hypochromasia (32.6%), microcytosis (14.7%) and macrocytosis (1.1%). The co-occurrence of malaria parasitaemia, iron deficiency and anaemia was seen in 23.3% of the women, iron deficiency anaemia only occurred in 35.9% while physiological anaemia of pregnancy was 40.9%. Iron deficiency and physiological anaemia of pregnancy contribute to a greater proportion of anaemia in the study area.

**Electronic supplementary material:**

The online version of this article (10.1186/s13104-017-2961-6) contains supplementary material, which is available to authorized users.

## Introduction

Studies have identified the haematological profile of the pregnant woman as one of the factors affecting pregnancy and its outcome [1, 2]. Low haemoglobin (anaemia) is the most widely identified haematological abnormality [3]. In malaria-endemic countries of Africa, approximately 50% of pregnant women are anaemic [4]. Anaemia in pregnancy represents a life-threatening but preventable cause of maternal and childhood morbidity and mortality [5].

The aetiological factors responsible for anaemia are multiple and their relative contributions vary by geographical area and by season [6]. Anaemia involves the complex interaction between nutrition, infectious disease (malaria, HIV, soil transmitted helminths particularly hook worm infestation), and other factors (socio-demographic and economic) [7]. Anaemia in pregnancy can be physiologic or pathologic. Physiological anaemia of pregnancy is a normal physiological phenomenon that occurs in pregnancy as a result of haemodilution [8, 9]. Physiological anaemia may turn into pathological anaemia in advanced pregnancy [10]. The principle of anaemia prevention in sub-Saharan Africa is the control of malaria and haematinics supplementation [11, 12]. Protection against malaria is usually achieved through the use of insecticide treated bed nets (ITNs), intermittent preventive treatment with sulphadoxine–pyrimethamine (IPTp-SP), prompt and effective case management of malaria [13].

Anaemia has been reported to invariably accompany infection with malaria parasite in endemic areas, though malaria parasitaemia may not be the primary cause of it [14]. It is thought that anaemia is mainly caused by iron deficiency (ID) in developing countries [4] and pregnant women are among the population groups at the highest risk [15]. Folic acid and vitamin B_12_ deficiencies or hemoglobinopathies may also contribute to increase risk of anaemia in pregnancy [16]. Other key determinants of anaemia include maternal age, parity levels, trimester of pregnancy, rural residents [16] and antenatal care [17].

Studies in the mount Cameroon area have shown consistently that anaemia is a severe (> 50%) health problem in pregnancy [18–20]. We hypothese that other causes than malaria parasitaemia account for a greater proportion of anaemia prevalence in pregnancy in this setting. In this study, haematological indices were measured and red blood cell abnormalities identified to assess possible causes of anaemia among pregnant women. This study reports on the prevalence of malaria related and non-malaria anaemia and its associated red blood cell anomalies at the time of the first antenatal clinic (ANC) visit.

## Main text

### Methods

#### Study area and population

This study was carried out in Mutengene and Muea Integrated Medical Centres in the Mt. Cameroon area. These centres are government owned institutions. Mutengene is a semi-urban town while Muea is a semi-rural setting. The characteristics of the study area have been described elsewhere [19]. IPTp-Sp and ITN usage in the area is associated with reduction in malaria parasitaemia and anaemia in pregnancy [20–22]. Pregnant women were recruited on their first antenatal visit and those who volunteered to participate in the study were enrolled consecutively.

#### Study design

This study is part of a prospective cross sectional study carried out in Mutengene and Muea from March to August 2013 [19]. At enrolment, a structured questionnaire was used to document socio-demographic characteristics (age, residence, and marital status), gynaecologic/obstetric history [gravidity status, gestational age (GA)] and socio-economic indicators (educational level, occupation, monthly income). Fever status (defined as temperature > 37.5 °C) was determined using a digital thermometer. Maternal peripheral venous blood (2 ml) was collected by venipuncture into ethylene diamine tetra-acetate (EDTA) tubes for haematological assessment and malaria parasite determination. All samples were transported on ice bath to the Malaria Research Laboratory, University of Buea for analysis.

*Parasitological analysis* Malaria parasitaemia was detected by microscopic examination of thin and thick blood films stained with 10% Giemsa. Slides were negative if no asexual parasites or gametocytes were seen after examining 100 high-power fields. Malaria parasitaemia was defined as the presence of any *Plasmodium* asexual stages (trophozoites and/or gametocytes) in blood films. Parasite density per microliter of blood was calculated per 200 leucocytes with reference to participants’ absolute WBC (white blood cell) [23].

*Haematological assessment* Blood samples were analysed for red blood cell indices: red blood cell (RBC) counts, Hb levels, haematocrit (Hct), MCV, MCH, mean corpuscular haemoglobin concentration (MCHC) and red cell distribution width—coefficient of variation (RDW-CV) following the manufacturer’s instructions using an automated haematology analyzer, the Beckman Coulter counter (URIT 3000).

*Definitions* Anaemia was defined as Hb < 11.0 g/dl [24]. Anaemia severity was as follows: mild anaemia (Hb: 10–10.9 g/dl), moderate anaemia (Hb: 7–9.9 g/dl), and severe anaemia (Hb < 7.0 g/dl) [24]. Microcytosis was defined as MCV less than 78 fl. Macrocytosis was defined as MCV more than 101 fl. Hypochromasia was defined as a MCH less 27 pg. Normocytic normochromic red cell morphology with haemoglobin concentration less than 11 g/dl in the absence of malaria parasitaemia was considered as physiological anaemia of pregnancy. Microcytic hypochromia or normocytic hypochromia with anaemia was considered as IDA.

#### Statistical analysis

Data were analysed using the statistical package for social sciences (SPSS package) version 0.20.

Descriptive statistics were computed for all relevant variables. Continuous variables (red blood cell parameters) were expressed as mean ± SD and compared between categorical variables (anaemic status, malaria-related and non-malaria anaemia, no malaria) using ANOVA and student’s paired t test. The Chi square test was used to assess the association between the red blood cell abnormalities and anaemia status. The level of statistical significance was set at p < 0.05.

### Results

#### Socio-demographic and economic characteristics

A total of 279 women enrolled were eligible for the study. The mean age (years) was 24.2 ± 5.2 (range 15–40 years). Majority of the women were married (68.7%; 189/275). The mean GA was 23.5 weeks (95% CI 22.8–24.2 weeks) and majority of women enrolled during the second and third trimesters (Table 1). The greater percentage of the women (61.6%; 159/258) reported a monthly income of less than 30.000 FCFA (franc des Communautés Financières d’Afrique) (approximately 60 USD) meanwhile 27.9% (72/258) and 10.5% (27/258) had an income between 30,000–50,000 (60–100 USD) and 50,000–75,000 CFA (100–150 USD) respectively. About 50% (49.3; 135/274) of the women had at most a secondary level of education.

**Table 1:** Clinical characteristics of the study participants

Variables	Categories	n	%
Age groups (279)	≤ 20	80	28.7
	21–25	95	34.1
	25	104	37.3
Gravidity groups (276)	Primigravidae	74	26.8
	Secundigravidae	94	34.1
	Multigravidae	108	39.1
Trimester of first ANC (277)	First	9	3.2
	Second	152	54.9
	Third	116	41.9

	N	Mean ± SD	Interquartile range	
Hb levels (µg/dl)	279	10.7 ± 1.6	9.7	11.9
Hct	279	32.8 ± 4.5	30	35.3
MCV (fl)	279	85.8 ± 7.8	82	90
MCH	279	27.8 ± 3.1	26	30
MCHC	279	32.4 ± 1.7	31.4	33.6
RBCs × 10^12^/l	279	3.9 ± 0.6	3.6	4.1
RDW-CV (%)	279	15.6 ± 2.3	14.1	16.4
Geometric mean parasite density (µl) (GMPD)	61	533.1	29.6	18,570.6

	n	%
Anaemia prevalence	159	57
Anaemia severity		
Severe	4	1.4
Moderate	83	29.7
Mild	72	25.8
Microcytosis	41	14.7
Macrocytosis	3	1.1
Hypochromasia	91	32.6
Malaria parasite infection (N = 279)	61	21.9
Malaria prevalence (N = 238)	36	15.1

#### Clinical characteristics and red blood cell alterations of pregnant women at first ANC clinic

The quantitative blood profile of the women indicates that the mean RBC count, MCV, MCH, MCHC and RDW-CV were within normal range, while the mean Hb concentration and Hct were lower than normal. Anaemia was the most frequent abnormality (Hb < 11.0 g/dl) of which majority were moderate to mild. The most frequent red blood cell alteration was hypochromasia followed by microcytosis. Macrocytosis was rare. Among the women with recorded temperature readings (N = 238), 15.1% with *P. falciparum* infection developed malaria while 10.1% (n = 25) had asymptomatic parasitaemia (Table 1).

#### Malaria-related and non-malaria anaemia and haematological indices

Among the anaemic cases, 23.3% (37) had malaria parasitaemia while 76.7% (122) cases were aparasitaemic. A total of 120 (43%) women were non-anaemic. Haemoglobin levels strongly (p < 0.001) correlated with MCH (r = 0.425), MCV (r = 0.436), MCHC (r = 0.522), MCV (r = 0.214), RBC (r = 0.506), Hct (r = 0. 621) and RBCDW-CV (r = − 0.366) levels. Generally, anaemia was associated with lower levels of MCH, MCHC, Hct, RBC counts, and higher RDW-CV levels when compared with non-anaemic individuals (Table 2). Nonetheless, red blood cell indices were comparable between malaria-related and non-malaria anaemia with the exception of MCV. Non-malaria anaemia and not malaria-related anaemia was associated with a decrease in MCV levels when compared with no anaemia.

**Table 2:** Comparison of mean levels of Hb MCH, MCHC, MCV, RBCs, Ht and RDW-CV among anaemic cases, malaria-related, non-malaria and non-anaemic cases

Anaemic status/red blood cell indices	Hb (g/dl)	MCH (pg)	MCHC (g/dl)	MCV (fl)	RBC (× 10^12^/l)	Ht (%)	RDW-CV (%)
^$^All anaemic cases	9.6 ± 1.0	26.8 ± 3.4	31.8 ± 1.6	84.5 ± 8.9	3.6 ± 0.5	29.9 ± 3.3	16.2 ± 2.5
*Malaria-related anaemia (n = 37)	9.5 ± 1.2	27.7 ± 3.6	32.0 ± 1.3	86.7 ± 10.3	3.5 ± 0.5	29.5 ± 2.7	16.0 ± 1.8
^&^Non-malaria anaemia (n = 122)	9.6 ± 1.0	26.3 ± 3.2	31.7 ± 1.7	83.8 ± 8.4	3.6 ± 0.5	30.1 ± 3.4	16.2 ± 2.7
^#^Non-anaemic (n = 120)	12.2 ± 0.9	29.2 ± 2.1	33.4 ± 1.3	87.7 ± 5.6	4.2 ± 0.4	36.4 ± 2.8	14.9 ± 1.9

Statistical significant difference between ^$^ and ^#^: Hb (t = 21.5; p < 0.001), MCH (t = 6.9; p < 0.001), MCHC (t = 9.1; p < 0.001), MCV (t = 3.5; p = p < 0.001) RBC (t = 10.7; p < 0.001), Hct (t = 17.4; p < 0.001), RDW-CV (t = 5.0; p < 0.001) levels

No statistical significant difference between * and ^&^: Hb (t = 0.8; p = 0.42), MCH (t = 1.9; p = 0.07), MCHC (t = 1.0; p = 0.33), MCV (t = 1.7; p = 0.09), RBC (t =1.7; p = 0.09), Hct (t = 0.9; p = 0.36) and RDW-CV (t = 0.5; p = 0.65) levels

Statistical significant difference between * and ^#^: Hb (t = 14.8; p < 0.001), MCH (t = 3.2; p = 0.002), MCHC (t = 5.7; p < 0.001), RBC (t = 8.5; p < 0.001), Hct (t = 13.1; p < 0.001), RDW-CV (t = 3.6; p < 0.001) except MCV (t = 0.7; p = 0.45) levels

Statistical significant difference between ^&^ and ^#^: Hb (t = 20.2; p < 0.001), MCH (t = 7.5; p = 0.002), MCHC (t = 8.7; p < 0.001), MCV (t = 4.2; p = p < 0.001) RBC (t = 9.6; p < 0.001), Hct (t = 15.9; p < 0.001), RDW-CV (t = 4.7; p < 0.001) levels

#### Association of red blood cell morphologies, haematological indices and anaemia status

**Fig. 1:**
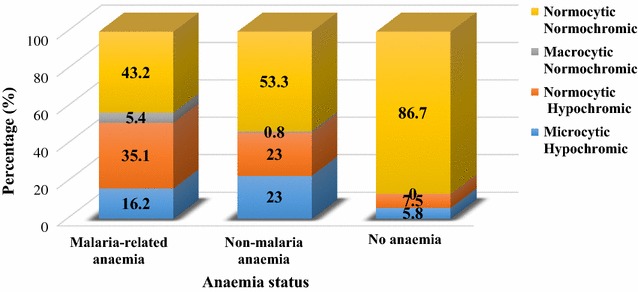
Distribution of the different red blood cell morphologies among malaria-related, non-malaria and non anaemic women

A greater proportion (66.3%; n = 185) of women had normal red blood cell morphology (normocytic normochromic) followed by normocytic hypochromia (17.9%; n = 50), microcytic hypochromia (14.7%) and macrocytic normochromia (1.1%). Compared with women with normocytic normochromic red cell morphology, those with microcytic hypochromic, macrocytic normochromic and normocytic hypochromic red cell profile had significantly lower haemoglobin levels. Microcytic hypochromia was associated with lower MCV and MCH levels while normocytic hypochromia was linked to lower MCH but normal MCV levels (Additional file 1). The proportions of red blood cell morphologies differed significantly (χ^2^ = 48.7; p < 0.001) among malaria-related, non-malaria and no anaemia (Fig. 1). A higher proportion of normocytic hypochromia was associated with malaria-related anaemia while microcytic hypochromia and normocytic hypochromia were equally related non-malaria anaemia.

#### Possible causes of anaemia among pregnant women at first ANC clinic in the study area

Among the women, the proportion of microcytic hypochromic and normocytic hypochromic anaemia was 12% (34) and 14.7% (41) respectively giving an overall prevalence of 26.9% as IDA.

Macrocytic anaemia constituted 1.1% while normocytic normochromic anaemia was 29% (81). The co-occurrence of malaria parasitaemia, iron deficiency and anaemia was seen in 37 (23.3%) whereas IDA only occurred in 57 (35.9%). Physiological anaemia of pregnancy was observed in 65 (40.9%). The prevalence of anaemia at ANC enrollment did not differ among the maternal age groups, gravidity status, trimester of pregnancy nor socioeconomic status.

### Discussion

A high prevalence of anaemia (57%) was observed in this study which is within the prevalence range (35.0–75.0%) in developing countries [25]. Anaemia prevalence was similar with findings from semi-urban settings in Nigeria (54.5%) [26] and Ethiopia (56.8%) [27] but lower than that observed in some rural areas of Demographic Republic of Congo (DRC) (61.1%) [28] and Benin (68.3%) [29, 30]. About 23% of anaemia was related to malaria parasitaemia. Previously, Achidi et al. [18] reported anaemia prevalence of 68.9% at ANC enrollment in the Mt. Cameroon area of which 52.1% of anaemia was malaria related. No association was observed between *P. falciparum* infection and Hb levels and anaemia suggesting falciparum infection may not be a significant contributing factor of anaemia among pregnant women in this area.

The most common red blood cell alterations were normocytic hypochromia and microcytic hypochromia which are indicators of iron deficiency. It is assumed that half of all cases of anaemia are caused by iron deficiency. Equally about 27% of anaemic cases observed was due to iron deficiency. Iron deficiency may due to inadequate diet, infection, or frequent pregnancies occurring shortly after one another [31]. Low socio-economic status of the women may contribute to the level of IDA observed in the present study. Financial limitations may hinder access to a diet high in digestible forms of iron such as animal proteins and dietary supplementation (more expensive mineral and vitamins rich-foods) [32–34] as well as may delay early initiation of ANC and uptake of anaemia preventive treatment.

Aparasiteamic women with normocytic normochromic anaemia may be experiencing physiological anaemia of pregnancy. However, this warrants further investigation. Most women in this setting enrolled for ANC in the second and third trimester of pregnancy. During this period the haemodilutional effect of pregnancy and increased foetal demand for haematopoietic factors are maximal [35]. The co-occurence of malaria parasitaemia, iron deficiency and anaemia may indicate anaemia of inflammation. Underlying maternal diseases such as malaria and nutritional deficiency in early pregnancy are likely to worsen anaemia course in pregnancy if not treated or prevented. In accordance with this, malaria—related and non-malaria anaemia were associated with significant lower levels of haematological indices. Anaemia of inflammation by malaria parasitemia induces changes in iron absorption and distribution resulting in prolonged sequestration of iron into storage forms limiting maternal use and potentially inhibiting delivery of iron to the foetus [36].

## Limitations

- Peripheral blood microscopy is a less sensitive method compared with molecular techniques [real-time polymerase chain reaction (PCR)], placental blood microscopy and histology. It is possible that peripheral parasitaemia may remain below the levels of microscopic detection [37].
- The examination of stained bone marrow aspirate for haemosiderin is the gold standard for the diagnosis of iron deficiency anaemia but this method is invasive [26]. Serum ferritin measurement on the other hand is costly.
- Helminth infestations, genital and urinary infections are associated with an increased risk for anaemia.
- Haemoglobinopathies particularly α+-thalassaemia heterozygote individuals may be at risk of anaemia [16].

## Additional file

**Additional file 1.** Comparison of mean levels of Hb MCH, MCHC, MCV, RBCs, Ht and RDW-CV among women with different red blood cell morphologies. This file describes the mean levels of Hb, MCH, MCHC, MCV, RBCs, Ht and RDW-CV among pregnant women with microcytic hypochromic, macrocytic normochromic, normocytic hypochromic and normocytic normochromic red blood cell morphologies.

## Abbreviations

ANC: Antenatal clinic
GA: Gestational age
Hb: Haemoglobin
Hct: Haematocrit
IPTp-SP: Intermittent preventive treatment with sulphadoxine-pyrimethamine
IDA: Iron deficiency anaemia
ITN: Insecticide treated net
MCH: Mean corpuscular haemoglobin
MCV: Mean corpuscular volume
MCHC: Mean corpuscular haemoglobin concentration
RBC: Red blood cell
RDW-CV: Red cell distribution width—coefficient of variation
WBC: White blood cell
