# Supplementary material for: Profile of red blood cell morphologies and causes of anaemia among pregnant women at first clinic visit in the mount Cameroon area: a prospective cross sectional study
Source: BMC Res Notes. 2017 Nov 29;10:645. doi: 10.1186/s13104-017-2961-6 (PMC5707787; doi:10.1186/s13104-017-2961-6)
Supplement: Supplementary file 1 — Additional file 1. Comparison of mean levels of Hb MCH, MCHC, MCV, RBCs, Ht and RDW-CV among women with different red blood cell morphologies. This file describes the mean levels of Hb, MCH, MCHC, MCV, RBCs, Ht and RDW-CV among pregnant women with microcytic hypochromic, macrocytic normochromic, normocytic hypochromic and normocytic normochromic red blood cell morphologies. [file 13104_2017_2961_MOESM1_ESM.docx]

**Additional file 1: Comparison of mean levels of Hb MCH, MCHC, MCV, RBCs, Ht and RDW-CV among women with**

**different Red blood cell morphologies**

| **Red blood cell morphology/ indices** | **Hb (g/dl)** | **MCH (pg)** | **MCHC (g/dl)** | **MCV**± **(fl)** | **RBC**  **(× 10^12^/l)** | **Ht (%)** | **RDW-CV (%)** |
| --- | --- | --- | --- | --- | --- | --- | --- |
| **Microcytic**  **Hypochromic**  **(41)** | 9.4 ± 1.7 | 22.4 ± 2.2 | 30.8 ± 1.6 | 72.6 ± 5.0 | 4.1 ± 0.6 | 30.2 ± 4.5 | 17.8 ± 3.1 |
| **Normocytic**  **Hypochromic**  **(50)** | 9.8 ± 1.2 | 25.9 ± 1.0 | 31.3 ± 1.3 | 82.8 ± 2.7 | 3.8 ± 0.5 | 31.0 ± 4.6 | 16.3 ± 2.4 |
| **Macrocytic**  **Normochromic**  **(3)** | 9.5 ± 1.1 | 34.4 ± 4.9 | 30.8 ± 1.9 | 111.5 ± 9.5 | 2.8 ± 0.7 | 30.9 ± 5.1 | 17.8 ± 2.3 |
| **Normocytic**  **Normochromic**  **(185)** | 11.2 ± 1.5 | 29.4 ± 1.5 | 33.1 ± 1.3 | 89.2 ± 4.5 | 3.8 ± 0.5 | 33.8 ± 4.1 | 14.9 ± 1.6 |
| **All cases** | 10.7 ± 1.6 | 27.8 ± 3.1 | 32.4 ± 1.7 | 85.8 ± 7.8 | 3.9 ± 0.5 | 32.7 ± 4.5 | 15.6 ± 2.3 |
| **Significant difference** | *F = 24.9  P < 0.001 | F = 258.5  P < 0.001 | F = 44.8  P < 0.001 | F = 206.5  P < 0.001 | F = 8.8  P < 0.001 | F = 12  P < 0.001 | F = 28.1  P < 0.001 |

* ANOVA Test
